# MiMIF-2 Effector of *Meloidogyne incognita* Exhibited Enzyme Activities and Potential Roles in Plant Salicylic Acid Synthesis

**DOI:** 10.3390/ijms21103507

**Published:** 2020-05-15

**Authors:** Jianlong Zhao, Zhenchuan Mao, Qinghua Sun, Qian Liu, Heng Jian, Bingyan Xie

**Affiliations:** 1 Institute of Vegetables and Flowers, Chinese Academy of Agricultural Science, Beijing 100081, China; zhaojianlong@caas.cn (J.Z.); maozhenchuan@caas.cn (Z.M.); sunqinghua_h@163.com (Q.S.); 2 Department of Plant Pathology and Key Laboratory of Plant Pathology of the Ministry of Agriculture, China Agricultural University, Beijing 100193, China; liuqian@cau.edu.cn

**Keywords:** *Meloidogyne incognita*, effector, MiMIF-2, enzyme activity, transcriptome, salicylic acid

## Abstract

Plant-parasitic nematodes secrete a series of effectors to promote parasitism by modulating host immunity, but the detailed molecular mechanism is ambiguous. Animal parasites secrete macrophage migration inhibitory factor (MIF)-like proteins for evasion of host immune systems, in which their biochemical activities play essential roles. Previous research demonstrated that MiMIF-2 effector was secreted by *Meloidogyne incognita* and modulated host immunity by interacting with annexins. In this study, we show that MiMIF-2 had tautomerase activity and protected nematodes against H_2_O_2_ damage. *MiMIF-2* expression not only decreased the amount of H_2_O_2_ generation during nematode infection in *Arabidopsis*, but also suppressed Bax-induced cell death by inhibiting reactive oxygen species burst in *Nicotiana benthamiana*. Further, RNA-seq transcriptome analysis and RT-qPCR showed that the expression of some heat-shock proteins was down regulated in *MiMIF-2* transgenic *Arabidopsis*. After treatment with flg22, RNA-seq transcriptome analysis indicated that the differentially expressed genes in *MiMIF-2* expressing *Arabidopsis* were pointed to plant hormone signal transduction, compound metabolism and plant defense. RT-qPCR and metabolomic results confirmed that salicylic acid (SA) related marker genes and SA content were significantly decreased. Our results provide a comprehensive understanding of how MiMIF-2 modulates plant immunity and broaden knowledge of the intricate relationship between *M. incognita* and host plants.

## 1. Introduction

Root-knot nematodes (RKNs; *Meloidogyne* spp.) are one of the most destructive obligate parasites of plants, infecting more than 5500 plant species and leading to over 70 billion dollars losses annually [1,2]. After hatching, pre-parasitic juveniles are attracted by the roots, infect, parasitic juveniles enter the vascular organization, choose feeding sites, induce to form ‘giant cells’ and molt three times [3] and complete a generation in some weeks. While plants are never passive, they have evolved to possess innate immunity to survive from various attacks. In recent decades, there are many thought-provoking researches, which give us inspirations on plant and RKNs interaction. In 2006, researchers put forward the ‘zigzag’ model to elucidate the interaction between pathogens and plants, which indicating the competitive state between pathogens and hosts [4]. In plant cells, the first layer immunity is pathogen-associated molecular pattern triggered immunity (PTI) that based on plant cell surface receptors. The second layer immunity is the recognition of pathogen effectors by plant resistance proteins, which is called effector-triggered immunity (ETI) [5]. Recently, researchers proposed to define plant immunities based on microbe recognition—either extracellular or intracellular, which is called “spatial immunity model” [6]. This model is widely accepted as depicting immune signaling during plant–microbe interactions. As successful biotrophic pathogens, RKNs have adapted to fine-tune host immune responses in an evolutionary arms race, of which a mass of secretions play essential roles in modulating plant immunity [7,8,9,10].

In the light of research evidence, plants recruit complex phytohormone signaling networks to defend pathogens, especially during ETI [11,12]. Although it is not clear what is the criterion of a nematode feeding site (NFS) and how to form giant cells (GCs) or syncytium, plant parasitic nematodes manipulate phytohormone pathways for NFS construction and GCs/syncytium formation [13]. Evidence showed that the level of plant SA is elevated in reaction with PTI and ETI [14,15]. Molecular mechanism investigation provided evidence that the expression of some *PR* (pathogenesis-related) genes depended on SA, which encoded proteins with anti-microbial activities [16]. Regarding this, fungal and oomycete pathogens secreted effectors (for example, chorismate mutase and isochorismatase) to promote infection by modulating SA biosynthesis [17,18]. Likewise, plants accumulated lower SA levels showed more susceptible to plant-parasitic nematodes [19,20], whereas enhanced SA levels showed less nematode infections [13,21]. Although large quantities of RKNs effectors were shown to suppress plant immune responses [10], only few effectors have potential links involving in modulating SA-mediated defense [12].

Reactive oxygen species (ROS) are involved in many biologic processes. For example, they modulate signal transduction in cells and plant development, response to biotic and abiotic stresses and relate to programmed cell death (PCD) [22,23,24,25]. The ROS signaling network is very conserved in plants, which integrates ROS producing pathways and ROS scavenging mechanisms [26]. Evidence showed that ROS burst were triggered when bacterial, fungal or viral pathogens recognized by plant host [25]. Likewise, ROS burst was also triggered by RKNs and cyst nematodes infection, which was modulated by plant NADPH oxidases to limit plant cell death and promote parasitism [27]. Moreover, evidence showed that RKNs secreted effectors to fine-tune ROS burst. MjTTL5 was an effector of *Meloidogyne javanica*. MjTTL5 interacted with *Arabidopsis* ferredoxin: thioredoxin reductase catalytic subunit (AtFTRc), which was involved in host antioxidant system [28]. Accumulated evidence suggested that ROS was integrated with plant hormone signaling pathways to regulate plant processes and response to environmental factors, of which ROS triggered SA increase and SA subsequently enhanced ROS accumulation for plant immunity [29,30].

Macrophage migration inhibitory factor (MIF) like proteins are multi-functional proteins, which is regarded as a major regulator of innate and adaptive immune responses [31,32]. Evidence showed that biologic and enzymatic activities of MIF-like proteins played vital roles [32]. Previous research confirmed that Human MIF recombinant protein possessed phenylpyruvate tautomerase *in vitro*, which *ρ*-hydroxyphenylpyruvate and phenylpyruvate were substrates [33]. However, there was no report about the endogenous substrates for MIF tautomerase activity. MIF-like proteins from *Helminths* and *Protozoa* were shown to play crucial roles in modulating host immune responses and their enzyme activities were investigated. For instance, *Bm*MIF was proved to secret by the filarial parasite *Brugia malayi,* which modulated host immune system and possessed tautomerase activity [34]. There were four MIF-like proteins in the free-living nematode *Caenorhabditis elegans*, they all had tautomerase activity and three of four showed oxidoreductase activity [35]. Parasites usually use sophistic means by which they successfully escape from host immune attack. *Trichinella* was reported to produce MIF that evaded host immunity and exhibited tautomerase and oxidoreductase activities [36,37]. *Plasmodium falciparum* is known for infamous malaria. *P*MIF was secreted by *Plasmodium*, which possessed tautomerase and oxidoreductase activities [38]. Recent report confirmed that the MiMIF-2 was a MIF-like protein and expressed in the *M. incognita* hypodermis and secreted into plant tissues. Moreover, MiMIF-2 modulated host immunity by interacting with two *Arabidopsis* annexins [39]. However, the enzyme activities of MiMIF-2 were not investigated. And it is fascinating to study whether MiMIF-2 possesses tautomerase and oxidoreductase activity during interaction with plant host cells.

In this study, we showed that MiMIF-2 exhibited tautomerase activity and played roles in protecting nematodes from oxidative stress, which was in accordance with the function of mammalian MIF-like proteins [40]. Transcriptomic profiling and hormone quantification revealed that *MiMIF-2* expression in *Arabidopsis* not only affected SA-mediated defense pathway, which was an essential signaling pathway in plants [14,30,41,42], but also interfered phenylpropanoid pathway after treatment with flg22. Together, these results provided a comprehensive interpretation that how MiMIF-2 effector affected plant immunity after entering host cells. Moreover, this work could broaden our knowledge to the multiple functions of *M. incognita* effector MiMIF-2, which integrated with host ROS, SA and compound metabolism for promoting parasitism.

## 2. Results

### 2.1. Tautomerase Activity of MiMIF-2

**Figure 1:**
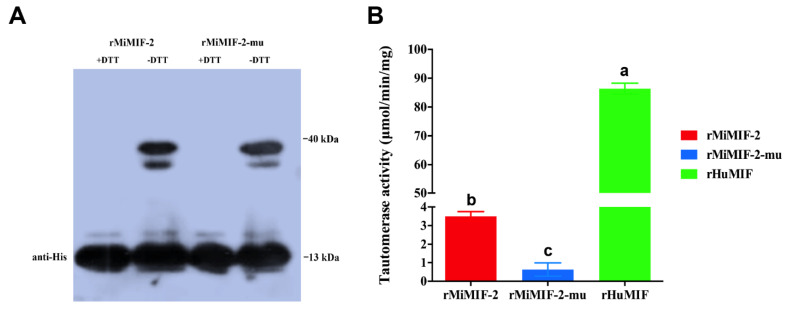
Recombinant MiMIF-2 exhibited tautomerase activity; (**A**) Detection of the purified recombinant rMiMIF-2 and rMiMIF-2-mu with an anti-His tag antibody. rMiBMIF-mu is the mutant in which the N-terminal proline is replaced with a glycine residue; (**B**) The tautomerase activities of rMiMIF-2 and its mutant were compared with that of the recombinant human MIF (rHuMIF), with p-hydroxyphenylpyruvate as the substrate. The bar represents the mean ± SD of five independent measurements. Columns marked with different letters were significantly different according to ANOVA followed by Dunnett’s multiple comparisons test (Alpha 0.05). More than three technical replicates and three biologic replicates were performed, and we show the results of one of these experiments here.

To identify the possible tautomerase activity of MiMIF-2, the recombinant MiMIF-2 with a N-terminal His tag (rMiMIF-2) and a mutated version with a glycine in place of the first proline residue (rMiMIF-2-mu) were used. These proteins were purified from the cells and checked by western blotting. In reducing conditions (+DTT, Dithiothreitol), the His tag antibody recognized the soluble purified rMiMIF-2 and rMiMIF-2-mu proteins at the expected size (13 kDa). After non-reducing (-DTT) electrophoresis, the molecular mass of the purified recombinant proteins was approximately 40 kDa, indicating the formation of a trimer (Figure 1A). The rMiMIF-2 catalyzed the tautomerization of the *p*-hydroxyphenylpyruvate substrate, but its activity was much lower than that of human MIF (Figure 1B). Moreover, rMiMIF-2-mu had only residual activity toward the substrate (Figure 1B). These results indicated that rMiMIF-2 had tautomerase activity.

### 2.2. MiMIF-2 Has Antioxidant Activity

**Figure 2:**
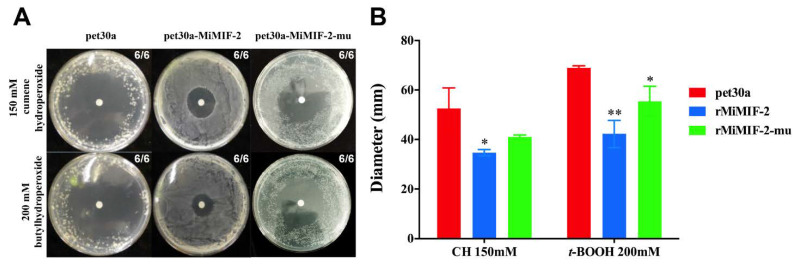
MiMIF-2 expression in *Escherichia coli* exhibited resistance to organic peroxide; (**A**) Sensitivity to cumene hydroperoxide (CH) or *t*-butyl hydroperoxide (*t*-BOOH), measured in the filter disc assay in *Escherichia coli* expressing MiMIF-2 or MiMIF-2-mu. Cells containing the empty plasmid pet30a were used as controls. The numbers indicate summary statistic data for Petri dishes with similar results. The experiments were performed three times; (**B**) Diameters were measured to assess the difference between treatments. The bar represents the mean ± SD of six independent measurements. *, ** indicates significant difference in one-way ANOVA with Dunnett′s multiple comparisons test (Alpha 0.05). The experiment was repeated three times with similar results.

To examine whether MiMIF-2 exhibited redox activity, *Escherichia coli* (*E. coli*) expressing rMiMIF-2 was challenged with organic peroxides (cumene hydroperoxide, CH and t-butyl hydroperoxide, *t*-BOOH). As shown in the result, MiMIF-2 expression significantly increased the tolerance of *E. coli* to CH and *t*-BOOH (Figure 2A), as measured by a 65% and 60% reduction in the diameter of the dead bacteria, respectively, relative to cells transformed with empty vector controls (Figure 2B). Likewise, rMiMIF-2-mu also increased the tolerance of *E. coli*, but to a less extent than rMiMIF-2 (Figure 2).

**Figure 3:**
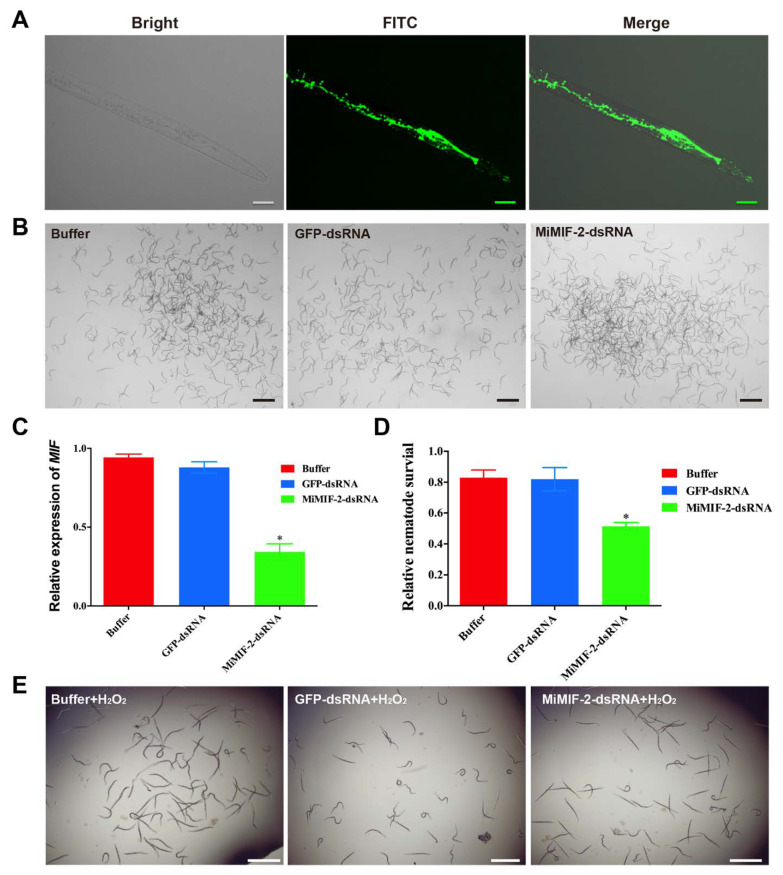
In vitro RNAi silencing of *MiMIF-2* in pre-parasitic *M. incognita* J2; (**A**) Fluorescence microscopy investigation showed ingestion of fluorescein isothiocynate (FITC) in the treated *M. incognita* pre-J2 s. Scale bars, 20 µm; (**B**) phenotypes of *M. incognita* pre-J2 s after treatment with buffer, GFP-dsRNA or MiMIF-2-dsRNA. Scale bars, 50 µm; (**C**) relative expression levels of *MiMIF-2* after soaking in the buffer, GFP-dsRNA or MiMIF-2-dsRNA. The relative expression of *MiMIFs* was quantified by RT-qPCR. The *tubulin* gene was used as an internal control, and fold-change values (mean ± SE) were calculated relative to the buffer value by the 2^−ΔΔCt^ method. * indicates significant difference in one-way ANOVA with Dunnett′s multiple comparisons test (Alpha 0.05). The data shown are from one of three independent experiments; (**D**) MiMIF-2-dsRNA or buffer-treated nematodes were incubated with 10-mM H_2_O_2_. Living nematodes were counted after treatment 4 h. Asterisks indicate significant differences relative to the mean ±SD values for the control treatment in Student’s *t*-test (*p* < 0.05). The experiments were performed three times and three biologic replicates showed similar results; (**E**) phenotypes of nematodes after treatment with dsRNA and H_2_O_2_. Scale bars, 50 µm.

Further, in vitro RNAi experiment was conducted to investigate the role of *MiMIFs* in relieving oxidative stress. Compared with the treatment with buffer, there were no phenotypic changes in nematodes viability after treatment with *MiMIF-2* dsRNA or *GFP* dsRNA (Figure 3A,B). RT-qPCR performed using *MiMIF* specific primers (Table S1) demonstrated that *MiMIFs* transcript levels were decreased by 80%, whereas treatment with control *GFP* dsRNA had no effect (Figure 3C). Further, following treatment with 10-mM H_2_O_2_, nematodes viability was 30% lower in J2 s in which *MiMIF* genes silenced by RNAi than in those treated with *GFP* dsRNA or buffer (Figure 3D,E). Together, these results showed that MiMIF-2 was involved in antioxidant activity.

### 2.3. MiMIF-2 Suppresses Reactive Oxygen Species (ROS) Burst

**Figure 4:**
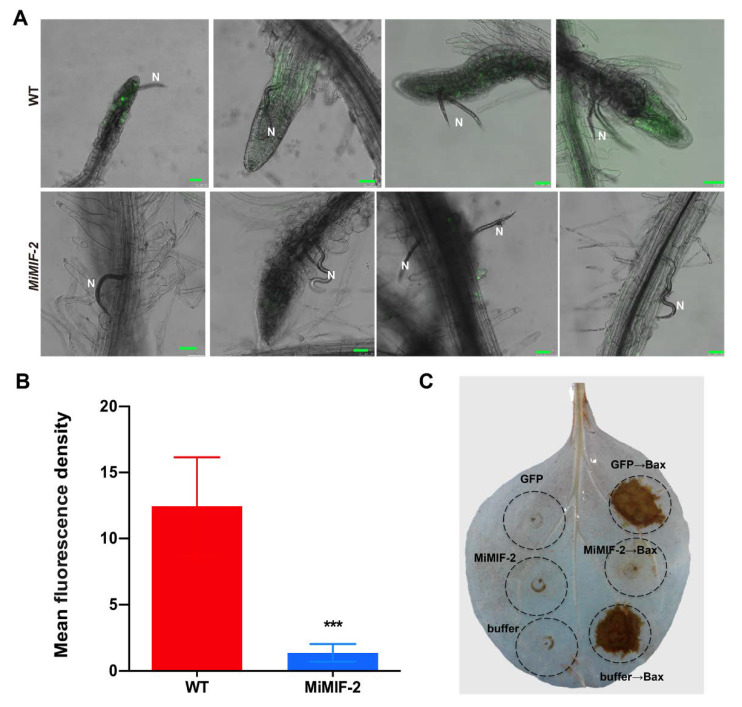
H_2_O_2_ visualization in nematode infected *Arabidopsis* roots and H_2_O_2_ detection after co-expression MiMIF-2 and Bax in *N. benthamiana* leaves; (**A**) CM-H_2_DCFDA staining of ROS in wild-type (WT) and *MiMIF-2* expressing (MiMIF-2-3 and MiMIF-2-4) roots after *M. incognita* infection. Images were captured by confocal microscopy (LeicaSP8) 1-day post inoculation (dpi). Scale bar, 50 µm. N, nematode; (**B**) Relative fluorescence density between wild type and *MiMIF-2* expressing *Arabidospsi* after *M. incognita* infection. *** indicates significant difference in the Student’s *t*-test (*p* < 0.001). (**C**) H_2_O_2_ production in *N. benthamiana* leaves was assessed by 3,3′-Diaminobenzidine (DAB) staining. *N. benthamiana* leaves were infiltrated with *Agrobacterium tumefaciens* carrying the corresponding constructs, and measurements were performed at 2 dpi. Three independent assays were conducted and at least 5 plants and/or 15 leaves were observed.

To investigate ROS production in plant roots upon nematode infection, *Arabidopsis* roots were labeled with 5-(and-6)-chloromethyl-2′,7′-dichlorofluorescein diacetate (CM-H_2_DCFDA) after *M. incognita* inoculation. Confocal microscopy observation results showed that, 1 day post infection (dpi) of *M. incognita*, there was significant difference (*p* < 0.001) of CM-H2DCFDA fluorescence intensity between wild type *Arabidopsis* and *MiMIF-2* expressing lines (Figure 4A and Figure S1), which decreased more than ten-fold in *MiMIF-2* expressing lines (Figure 4B).

To detect ROS generation during programmed cell death (PCD), MiMIF-2 and Bax were co-expressed in *N. benthamiana* leaves. *A. tumefaciens* cells carrying the *MiMIF-2* gene were infiltrated 24 h prior to infiltration with Bax, and two days post infiltration DAB staining was conducted to determine the amount of H_2_O_2_. The results showed that H_2_O_2_ production was significantly weak at the positions where MiMIF-2 was expressed (Figure 4C and Figure S2). Together, these results indicated that MiMIF-2 played roles in suppressing ROS burst *in planta*.

### 2.4. Identification of Differentially Expressed Genes (DEGs) in MiMIF-2 Expressing Arabidopsis Lines and Wild Type Plants

**Figure 5:**
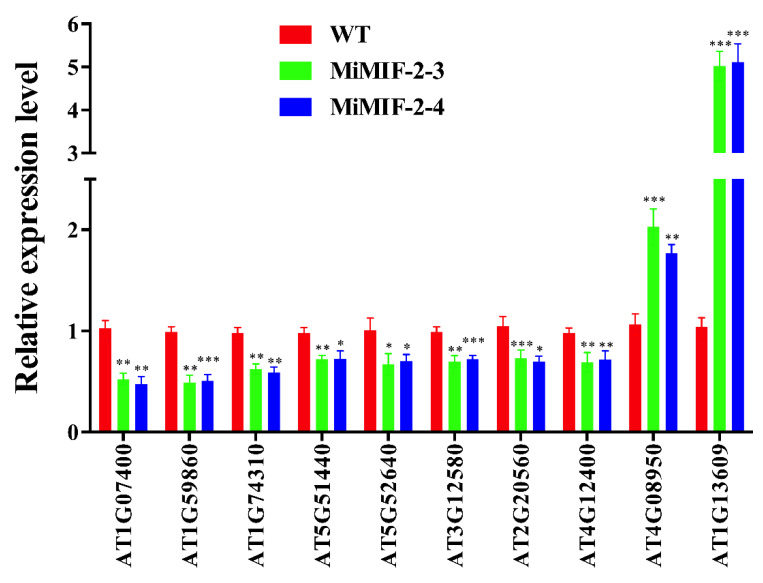
RT-qPCR verification of candidate differentially expressed genes (DEGs) between wild type and *MiMIF-2* expressing *Arabidopsis*. The relative expression levels of 8 heat-shock-like proteins and 2 upregulated genes were analyzed by RT-qPCR and normalized using *UBP22* (*AT5G10790*) as the internal control. Data represent the mean fold-change ± SE. Mean values significantly different from wild type (*, **, ***) were determined by one-way ANOVA with Dunnett′s multiple comparisons test (Alpha 0.05). The experiment was repeated three times with similar results.

We previously showed that ectopic expressing *MiMIF-2* in *Arabidopsis* showed identical phenotypes with wild type, but increased susceptibility to *M. incognita* [39]. To further investigate potential gene expression changes and signaling pathway modifications regulated by the MiMIF-2 effector, we conducted an RNA-seq experiment to compare wild type *Arabidopsis* and the MiMIF-2 transgenic lines. The FPKM method was used to calculate DEGs between WT and *MiMIF-2* ectopic expressing *Arabidopsis* lines. Under the criterion of |log_2_FC| ≥ 1 and FDR ≤ 0.1, compared with wild type, 26 DEGs were identified in *MiMIF-2* expressing *Arabidopsis* through pairwise comparisons (Data S1). Among them, 5 genes were upregulated and 21 genes were downregulated. Interestingly, the expression levels of 8 genes encoding heat-shock protein (HSP) associated proteins were downregulated (Data S1). In order to confirm the transcriptomic data, DEGs of all *HSP*-associated genes and two upregulated genes (*AT4G08950* and *AT1G13609*) were verified by RT-qPCR. On the whole, RT-qPCR showed that these 10 genes exhibited similar expression trends with the annotations of transcriptomic data, indicating that differential gene expression was due to the effect of the *MiMIF-2* expression (Figure 5).

### 2.5. Plant Response Pathways and Metabolism Pathways Were Interfered in MiMIF-2 Ectopic Expressing Arabidopsis after Treatment with flg22

**Figure 6:**
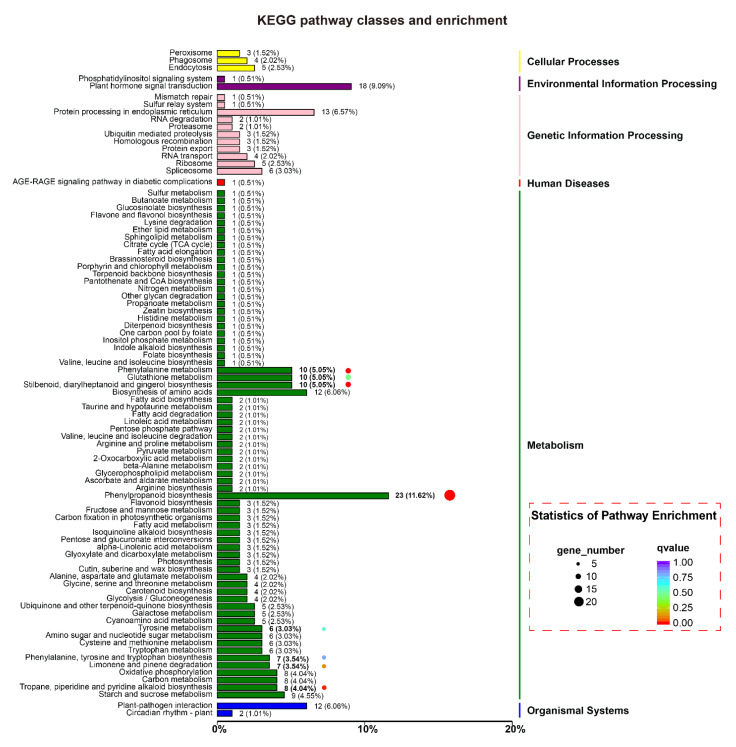
KEGG pathway enrichment of differentially expressed genes (DEGs) in *MiMIF-2* expressing *Arabidopsis* after treatment with flg22. DEGs were classified into 6 processes (cellular processes, environmental information processing, genetic information processing, human diseases, metabolism and organismal systems). *MiMIF-2* expressing in *Arabidopsis* affected some cellular pathways, especially phenylpropanoid biosynthesis, phenylalanine metabolism, glutathione metabolism, plant hormone signal transduction and plant–pathogen interaction.

Nematode infection induced ROS burst was significantly suppressed in *MiMIF-2* expressing *Arabidopsis*, indicating that *MiMIF-2* may interfere plant immunity pathways. In this study, transcriptomic profile on wild type and *MiMIF-2* ectopic expressing *Arabidopsis* was investigated after treatment with flg22 for 24 h. A total of 1204 DEGs were identified through pairwise comparisons in *MiMIF-2* ectopic expressing *Arabidopsis*, of which 438 genes were upregulated and 766 genes were downregulated (Data S2). COG function classification of these identified DEGs showed that they were categorized into 21 function classes. Among them, signal transduction mechanisms (14.89%), secondary metabolites biosynthesis, transport and catabolism (10.51%), carbohydrate transport and metabolism (8.23%), posttranslational modification, protein turnover, chaperones (7.53%), and defense mechanisms (6.65%) were relatively abundant (Figure S3A). Based on the three GO categories, the majority classifications of the DEGs were cellular process, metabolic process and single-organism process in “biologic process category”, cell part, cell and organelle in “cellular component category”, binding and catalytic activity in “molecular function category” (Figure S3B). And Kyoto Encyclopedia of Genes and Genomes (KEGG) enrichment analysis showed that 327 DEGs were mostly involved in metabolism, in which phenylpropanoid biosynthesis, phenylalanine metabolism, stilbenoid, diarylheptanoid and gingerol biosynthesis, tropane, piperidine and pyridine alkaloid biosynthesis were the main enriched pathways (Figure 6). This information suggested that *MiMIF-2* expression *in planta* mainly affected plant response pathways and metabolism pathways.

### 2.6. Defense-Associated Genes Were Down Regulated and Salicylic Acid (SA) Content Was Decreased in MiMIF-2 Expressing Arabidopsis after Treatment with flg22

**Figure 7:**
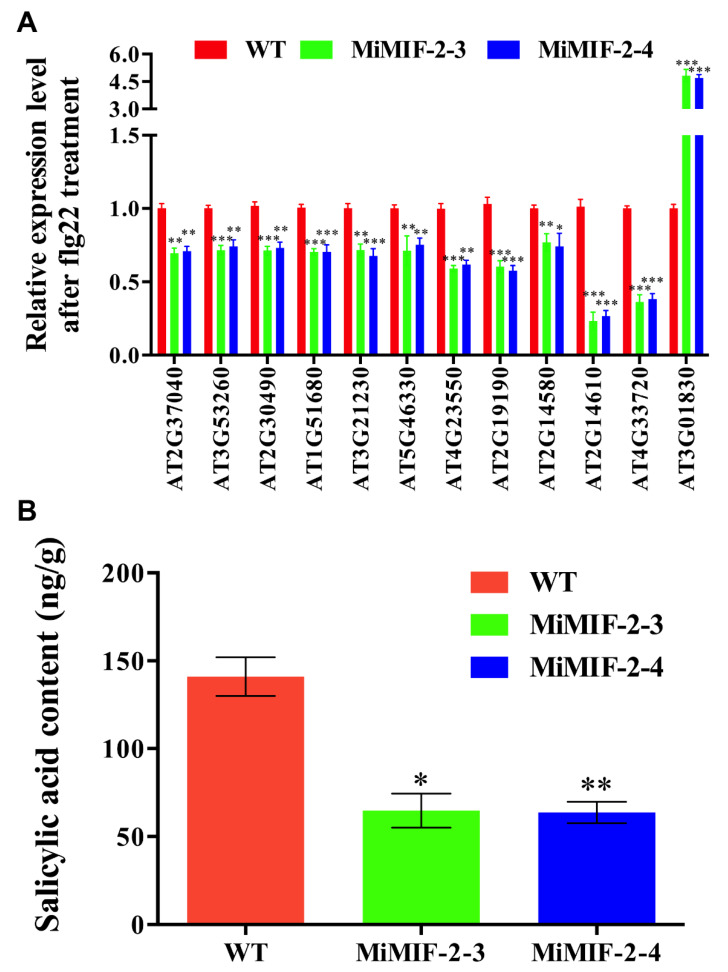
Candidate DEGs verification and metabolic analyses of SA content between wild type and MiMIF-2 expressing *Arabidopsis* after treatment with flg22 24 h; (**A**) The relative expression ratio of 12 genes involved in phenylpropanoid synthesis and plant–pathogen interaction were analyzed by RT-qPCR and normalized using *UBP22* (*AT5G10790*) as internal control. Data represent the mean fold-change ± SE. Mean values significantly different from wild type (*, **, ***) were determined by one-way ANOVA with Dunnett’s multiple comparisons test (Alpha 0.05). Three biologic repeats were performed with similar results; (**B**) SA content was quantified using seedlings of wild type and *MiMIF-2* expressing *Arabidopsis* after treatment with flg22 24 h. The bar represents the average of three independent measurements. *, ** indicating the significant difference from the wild type was determined in one-way ANOVA with Dunnett’s multiple comparisons test (alpha 0.05).

By analyzing the data of differentially expressed genes that involved in the pathways of plant–pathogen interaction, plant hormone signal transduction and phenylalanine metabolism, some defense-associated genes were down regulated, for example PR1, FRK1, WRK29 and PAL. Further, RT-qPCR was conducted to verify the expressions of key genes involved in defense pathways, phenylpropanoid biosynthesis and phenylalanine metabolism. And the results were in accordance with the transcriptome data, indicating that differential gene expression was due to the effect of the *MiMIF-2* expression (Figure 7A). Moreover, metabolic analyses revealed a dramatic decrease of SA content in *MiMIF-2* expressing *Arabidopsis* lines (Figure 7B), which suggested a potential effect of MiMIF-2 on the SA synthesis.

## 3. Discussion

MiMIFs are homologous to the human MIF and contain the conserved amino acids Pro1, Lys32 and Ile64, involved in tautomerase activity. Previous report demonstrated that the X-ray crystal structure of MIF was a homotrimer [43], which was related structurally to the small bacterial isomerases 4-oxalocrotonate tautomerase, 5-carboxy- methyl-2-hydroxymuconate and chorismate mutase [44]. Meanwhile, mammalian and helminth MIFs both have tautomerase activity [45]. In this study, our results showed that rMiMIF-2 formed a trimer in non-denaturing western blotting and catalyzed the tautomerization of *p*-hydroxyphenylpyruvate, but the activity was much weaker than that of HuMIF. A previous study indicated that the initiator methionine of MIF was cleaved, exposing the proline residue, which was essential for MIF tautomerase activity [46]. *Plasmodium falciparum*-derived MIF-like protein [47] and a MIF-like protein from the filarial parasite *O. volvulus* (OvMIF-2) had low levels of activity with the substrates *p*-hydroxyphenylpyruvate and *L*-dopamethylester [48]. Our findings confirmed the requirement for the N-terminal proline residue for tautomerase activity and raised the possibility that MIF may exert its biologic action via an enzymatic reaction.

MIF-like proteins have thiol–protein oxidoreductase activity in animals and this activity is dependent on a C-X-X-C motif. For instance, the human MIF can modulate immune responses through a cysteine-mediated redox mechanism [49]. Sequence analyses have shown that the C-X-X-C motif is not present in all helminth orthologs [45], but there is growing evidence for the involvement of MIFs in immune processes through a number of subtle mechanisms. *Plasmodium*-derived MIFs have two of the four cysteine residues of the CC motif and CXCR2 and CXCR4 have been identified as functional chemokine receptors [50]. In this study, the rMiMIF-2 protein could protect *E. coli* against organic peroxide and *MiMIF-2* dsRNA-treated nematodes were less viable in H_2_O_2_ solution than nematodes subjected to a control treatment. Interestingly, our result is similar with clade B *M. incognita* cuticle-secreted peroxiredoxin (PRX) proteins, which has protective anti-oxidant activity [51]. We speculate that MiMIF-2 may have evolved sophisticated functions in the redox system that may contribute to *M. incognita* parasitism.

ROS burst is a symbol of plant cell response to microbe infection. Bax is a death-promoting member of the Bcl-2 family of proteins, which triggers cell death when expressed in *N. benthamiana*. Previous evidence showed that Bax-induced cell death was similar to hypersensitive response and correlated with accumulation of the defense-related protein PR1 [52]. In this work, we co-expressed Bax with MiMIF-2 in *N. benthamiana* leaves. Results showed that H_2_O_2_ production was significantly alleviated after MiMIF-2 expression. Moreover, our nematode infection assay showed that CM-H2DCFDA fluorescence was weaker in *MiMIF-2* ectopic expressing lines than in the wild-type control. Together, these results indicated that MiMIF-2 probably facilitated parasitism by suppressing the host ROS burst.

Ectopic expression of *MiMIF-2* in *Arabidopsis* allowed us to identify the potential functions of *MiMIF-2* by assessing its effect on plant cells. We identified a total of 26 DEGs in response to *MiMIF-2* expression in *Arabidopsis* according to RNA-seq data. Interestingly, 8 DEGs encoded heat-shock proteins (HSPs) were all downregulated. These included HSP20, HSP70, HSP90 and some HSP family proteins. Only 5 DEGs were upregulated, of which three only had functional predication. Among them, *AT4G08950* encoded an *EXO* (*EXORDIUM*) gene, which was response to brassinosteroid (BR). Evidence showed that *EXO* overexpression promoted shoot and root growth, while *exo* knock out mutant lines showed reduced BR-triggered cotyledon and hypocotyl growth. Moreover, expression-profiling results indicated that EXO controlled a growth-related gene *expansion 5* [53]. Interestingly, plant expansins were involved in the establishment of feeding cells by plant-parasitic nematodes [54,55,56,57]. It would be meaningful to explore the interaction between EXO expression and *M. incognita* parasitism. Together, the transcriptome profile on wild type and *MiMIF-2* expressing *Arabidopsis* indicated that MiMIF-2 potentially involved in modulating *HSP*-like genes expression in plant cell, which could be an explanation for the abolished H_2_O_2_ production during nematode infection in *MiMIF-2* expressing *Arabidopsis* roots and *MiMIF-2*/Bax co-expression in *N. benthamiana* leaves.

Flg22 is a conserved peptide derived from *Pseudomonas syringae* flagellin. It is perceived by the receptor kinase FLS2 in *Arabidopsis*, leading to the induction of plant immune responses, such as callose deposition, the expression of defense marker genes, ROS burst and the activation of MAPK cascades [58,59,60,61]. Previous report showed that ectopic expressing *MiMIF-2* in *Arabidopsis* affected PAMP-triggered immunity, which MAPK cascade was interfered, some defense-related marker genes were significantly repressed and callose deposition was reduced after treatment with flg22 [39]. To further investigate the effect of *MiMIF-2* expression in plant cell in response to flg22, transcriptomic profile on wild type and *MiMIF-2* expressing *Arabidopsis* was investigated after treatment with flg22 for 24 h. Previous report indicated that transcriptional response to 1 µM flg22 was terminated within 24 Hours [62]. However, we identified 1204 DEGs, in which many genes were involved in plant–pathogen interaction. For instance, *AT5G46330* encodes FLS2, which is an essential leucine-rich repeat serine/threonine protein kinase involving plant innate immunity [63]. Strong evidence confirmed that flg22 was recognized by FLS2-BAK1 complex (BAK1 is a co-receptor) and activated downstream immune responses [64]. The FLS2 expression was inhibited in flg22 treated *MiMIF-2* expressing plants, which could affect downstream signal transduction and finally led to the suppression of immune-related marker genes expression. In accordance with the expression level of FLS2, *AT4G23550* encodes WRKY DNA-binding protein 29, which was shown to interact with the heat-shock factor f1b (hsf1b) involving abiotic stress tolerance [65]. Likewise, genes encoding FRK1 (*AT2G19190*) and pathogenesis-related proteins (*AT2G14580*, *AT2G14610* and *AT4G33720*) were all downregulated, of which *AT2G14610* encodes PR1 is essential for plant immunity and cell-to-cell signal transduction. Further metabolic analyses revealed that SA content was significantly decreased in *MiMIF-2* expressing *Arabidopsis* after treatment with flg22. Together, these results indicated that *MiMIF-2* expression may have a role in dampening plant responses to PAMP.

Based on the KEGG pathways of related DEGs, we noticed that phenylpropanoid biosynthesis and phenylalanine metabolism pathways were suppressed. Plant phenylpropanoids are involved in many cellular processes and functional pathways, especially plant growth and development, protection against biotic or abiotic stresses [66,67]. Starting from phenylalanine, phenylpropanoids pathway also produces a variety of different kinds of compounds, including SA. SA is an essential plant hormone against various biotic and abiotic stress, including restrict plant-parasitic nematode infection [13,30,41]. Although SA is predominantly synthesized via the chorismate/isochorismate pathway in *Arabidopsis* (about 90%), mutations in phenylalanine ammonia–lyase (PAL) genes resulted in a 90% reduction of basal PAL activity and 50% decrease in pathogen-induced SA accumulation, indicating the importance of PAL for SA biosynthesis [68]. A previous research demonstrated that the transcription of *PAL* gene was increased in resistant soybean cultivars after *M. incognita* infections [69]. Moreover, the expression of *PAL* gene was also activated in tomato when the plant was infected with *M. incognita* [70]. Phenylpropanoid biosynthetic pathway was also induced by pathogen infection [71,72]. *AT2G30490* encodes a cinnamate-4-hydroxylase (C4H), which catalyzes key reactions of phenylpropanoid pathways and modulates salicylic acid accumulation [73]. Recently, the biosynthetic pathways for phenylalanine and phenylalanine-derived compounds were activated for the induction of ETI [74], which gives inspirations for analyzing the role of phenylpropanoids during the interaction between plant-parasitic nematodes and hosts. In this study, we found some genes involved in phenylpropanoid pathways were downregulated, especially *AT2G37040* encodes PAL1, *AT3G53260* encodes PAL2, *AT2G30490* encodes C4H, and the decreased SA content in MiMIF-2 expressing *Arabidopsis*. Thus, we speculate that *MiMIF-2* expression in plant may interfere with host cell metabolism, especially the phenylpropanoid metabolic pathway, leading to endogenous hormone levels decreased.

In this study, we confirmed that MiMIF-2 had tautomerase activity and antioxidant activity. MiMIF-2 expression in *Arabidopsis* may affect plant defense pathways by suppressing SA synthesis. Altogether, our data suggested, to our knowledge, a possible molecular explanation for *M. incognita* MiMIF-2 effector, which interfered with plant cell metabolism and SA-mediated immune responses through enzymatic activities.

## 4. Materials and Methods

### 4.1. Nematode Collection and Plant Materials

*M. incognita* was reproduced on tomato plants (*Solanum lycopersicum* var. ‘Baiguo’). Infective pre-parasitic juveniles were collected after eggs hatching 24 h.

Two homozygous *MiMIF-2* expressing *Arabidopsis* lines (*MiMIF-2-T3-3* and *MiMIF-2-T3-4*) were used in this study, described previously [39]. Surface-sterilized *Arabidopsis* seeds were seeded on MS medium (Sigma, St. Louis, MO, USA) and placed in a plant growth incubator with a 16-h light and 8-h dark photoperiod at 23 °C. Tobacco (*Nicotiana benthamiana*) plants were grown in pots under the same condition with *Arabidopsis*.

### 4.2. SDS–PAGE, Immunoblotting and Biochemical Analyses of rMiMIF-2

Recombinant MiMIF-2 (rMiMIF-2, with an N-terminal His tag) was reported before [39]. SDS-PAGE was performed on a separating gel of 15% (*w*/*v*) acrylamide and a stacking gel of 4% (*w*/*v*) acrylamide. Protein was transferred to PVDF membrane using semi-dry transfer (Bio-Rad, Hercules, CA, USA). Western blot analysis was performed for the detection of rMiMIF-2 with His-tag antibody (Sigma, St. Louis, MO, USA).

The MiMIF-2 tautomerase assay was conducted as previously described [47]. Briefly, the substrate, *p*-hydroxyphenylpyruvate (Sigma, St. Louis, MO, USA), was dissolved in 50-mM ammonium acetate (pH 6.0) and the recombinant MIF protein was dissolved in boric acid (pH 6.2). The enzymatic activity was determined at 25 °C by addition of 50 µL of 0.2-mM *p*-hydroxyphenylpyruvate to a 96-well plate containing 50-µL MIF solution and measuring the increase in absorbance at 330 nm. Human MIF recombinant protein (AmyJet Scientific, Inc., Wuhan, China) was used as a positive control. This experiment consisted of three technical replicates; results were represented by the mean plus SD of three biologic replicates. The data were subjected to one-way ANOVA followed by Dunnett’s multiple comparisons test using Prism 6.0 (Alpha 0.05, GraphPad Software, Inc., La Jolla, CA, USA).

Bacteria viability assays were conducted as described previously [51]. Bacteria expressing rMiMIF-2 and rMiMIF-2-mu were transferred to Top agar (Luria–Bertani broth (LB) medium supplemented with 0.8% agar, 50-mg/mL kanamycin, 1-mM IPTG, Isopropyl β-D-thiogalactoside), plated onto LB agar plates (100 mg/mL kanamycin) and incubated for 1 h at 37 °C. Subsequently, filter discs (diameter 5 mm) soaked with 5 µL of 200-mM cumene hydroperoxide (CH) or 100-mM butyl hydroperoxide (*t*-BOOH) (Sigma, St. Louis, MO, USA) were placed on top of the agar. The diameter of the zone of killing was measured after an overnight incubation at 37 °C with 8 replicates for each condition. The mean plus SD value of 6 biologic replicates were presented. The data were subjected to one-way ANOVA with Dunnett’s multiple comparisons test. Three independent assays were performed.

### 4.3. In Vitro RNAi and Nematode Activity in H_2_O_2_

Vector construction was conducted according to previous research [75]. Briefly, *MiMIF-2* (339 bp) and fragments of green fluoresces protein (GFP) were cloned into the L4440 vector (a gift from Prof. Fanrong Zeng’s lab, Chinese Academy of Agricultural Sciences, Beijing, China) to construct L4440-MiMIF-2. Plasmids were transformed into HT115 competent cells and dsRNA was induced with IPTG. dsRNA was extracted using TRIzol and enzymatic digestion followed by DNase (TaKaRa, Shiga, Japan) and S1 nuclease (Invitrogen, Carlsbad, CA, USA), according to the manufacturer’s instructions. In vitro RNAi experiment was conducted following the previous protocol [76]. Freshly hatched *M. incognita* pre-J2s were soaked in solutions containing *MiMIF-2* dsRNA, *GFP* dsRNA or without dsRNA for 30 min. After soaking, the nematodes were used for RT-qPCR to quantity the interference effect and activity assay in 10-mM H_2_O_2_ (Sigma, St. Louis, MO, USA). Living nematodes were counted after treatment for 4 h. Each experiment was performed three times independently. Statistically significant differences between treatments were determined by one-way ANOVA with Dunnett’s multiple comparisons test.

### 4.4. Transient Expression Assay in N. benthamiana

To visualize H_2_O_2_, a type of reactive oxygen species (ROS), *N. benthamiana* leaves infiltrated with Bcl-2 associated X protein (Bax) and/or MiMIF-2 were stained with 3,3′-Diaminobenzidine (DAB). We used *Agrobacterium tumefaciens* cells to deliver a potato virus X (PVX) vector carrying *MiMIF-2* and a proapoptotic *Bax* ORF into *N. benthamiana* leaves. pGR107-MiMIF-2 and pGR107-Bax plasmids were reported before and *Agrobacterium* infiltration method was conducted as previously [39]. Generally, four-week-old *N. benthamiana* leaves were infiltrated with the recombinant *Agrobacterium* suspension (for each assay, five plants and /or 15 leaves were used for infiltration). Leaf samples were collected at 2 dpi and soaked in 1 mg/mL 3,3′-diaminobenzidine solution. The sampled leaves were placed in the dark for 4 h, boiled in 95% ethanol and photographed. This experiment was repeated at least three times with similar result.

### 4.5. Nematode Infection and Detection of Root Reactive Oxygen Species (ROS)

To monitor ROS production and localization in *Arabidopsis* root cells, the CM-H2DCFDA (C6827, Molecular Probes) staining method was used with some modifications of the previously described protocol [77,78]. Generally, lines expressing *MiMIF-2* or wild type *Arabidopsis* were grown under sterile conditions and inoculated with 200 surface-sterilized nematodes. The roots were then incubated with CM-H2DCFDA (10 μM) in phosphate-buffered saline for 90 min at 4 °C at 1 dpi. The samples were cleaned with KCl (0.1 mM) and CaCl_2_ (0.1 mM), kept at room temperature for 1 h, and then imaged with a Leica confocal microscope (Leica SP8, Heidelberg, Germany). Three independent assays were performed and at least 10 plants were observed for each line. Relative fluorescence density was calculated using ImageJ (NIH, Bethesda, MD, USA).

### 4.6. RNA Isolation, Library Construction and RNA-Seq

Thirteen-day-old *Arabidopsis* seedlings in sterile MS plates (WT and MiMIF-2 transgenic lines) were harvested. For flg22 treatment, seedlings were treated by 1 μM flg22 for 24 h in sterile Corning six-wells plate. *Arabidopsis* total RNA was isolated using the RNAsimple total RNA kit following the manufacturer’s instructions (Tiangen, Beijing, China). The quantity, quality and integrality of the extracted RNA were verified by Nanodrop (Wilmington, DC, USA), Qubit 2.0 (Invitrogen, Carlsbad, CA, USA) and Agilent 2100 (Agilent, USA). Libraries construction and the RNA-Seq were performed by the Biomarker Biotechnology Corporation (Beijing, China) using Illumina HiSeq2500. mRNA was enriched by oligo (dT)-rich magnetic beads and then broken into random fragments using Fragmentation Buffer. First- and second-strand cDNA were synthesized using these mRNA and purified by AMPure XP beads. The resulting cDNAs were then subjected to end-repair and add polyA tail. After that, Illumina sequencing adaptors were ligated to these cDNA and then use AMPure XP beads for fragment size selection. Next, the cDNA libraries were obtained by PCR enrichment. Finally, the cDNA libraries were sequenced using an Illumina HiSeq2500.

### 4.7. Data Analysis

Raw data (raw reads) of fastq format were first, processed through in-house Perl scripts. In this step, clean data (clean reads) were obtained by removing reads containing adapter, reads containing ploy-N and low-quality reads from raw data. At the same time, Q20, Q30, GC-content and sequence duplication level of the clean data were calculated. All the downstream analyses were based on clean data with high quality.

The adaptor sequences and low-quality sequence reads were removed from the data sets. Raw sequences were transformed into clean reads after data processing. These clean reads were then mapped to the reference genome sequence. Only reads with a perfect match or one mismatch were further analyzed and annotated based on the reference genome. Hisat2 tools soft were used to map with reference genome. To define the different expression genes (DEGs) between WT and *MiMIF-2* transgenic lines after treatment with flg22, the data were calculated based on the ratio of the RPKM values, and the false discovery rate (FDR) control method. In this study, data only with an value of log2 ratio ≥ 2 and an FDR significance score <0.01 were used for analysis. Without any treatment, comparison of WT and *MiMIF-2* transgenic lines used a value of log2 ratio ≥ 2 and a FDR significance score < 0.1.

Gene function was annotated based on the following databases: including the NCBI Nr (non-redundant protein sequences database), NCBI Nt (non-redundant nucleotide sequences database), Swiss-Prot (a manually annotated and reviewed protein sequence database), KEGG (the Kyoto Encyclopedia of Genes and Genomes, http://www.genome.jp/kegg/kegg2.html), Pfam (Protein family, http://pfam.xfam.org) and GO database (gene ontology, http://geneontology.org).

### 4.8. RT-qPCR Analysis

Real-time quantitative PCR (RT-qPCR) was performed on an ABI Prism 7000 real-time PCR system (Applied Biosystems, USA). Primers used in this study were listed in Table S1. RT-qPCR was performed using SYBR^®^ *Premix Ex Taq*™ II (TaKaRa, Japan) under the following program: 95 °C 30 s and 40 cycles of 95 °C for 5 s, 60 °C for 30 s and 95 °C, 15 s, 60 °C for 60 s and 95 °C for 15 s. Expression ratio was analyzed using the 2^−ΔΔCt^ method [79]. Data represent the mean fold-change ± SE. Statistical Analysis was used one-way ANOVA with Dunnett′s multiple comparisons test. This experiment was repeated three times.

### 4.9. Detection of Salicylic Acid (SA) Content

For metabolic analysis, wild type *Arabidopsis* seedlings and *MiMIF-2* transgenic *Arabidopsis* seedlings were treated with 1 µM flg22 for 24 h, and then immediately frozen in liquid nitrogen, ground into powder. The samples were extracted and quantified with an ABI QTRAP 6500 LC–MS/MS platform (Applied Biosystems, Foster City, CA, USA) according to previous method [80]. The standard SA was purchased from Sigma-Aldrich (St. Louis, MO, USA). Statistical Analysis was used one-way ANOVA with Dunnett′s multiple comparisons test.

### 4.10. Accession Number

Sequence data were stored at the National Genomics Data Center (NGDC). The raw sequence data reported in this study is stored at the Genome Sequence Archive (Genomics, Proteomics & Bioinformatics 2017) in BIG Data Center, Beijing Institute of Genomics (BIG), Chinese Academy of Sciences, under accession numbers CRA002198, which is publicly accessible at https://bigd.big.ac.cn/gsa.

## Supplementary Materials

Supplementary materials can be found at https://www.mdpi.com/1422-0067/21/10/3507/s1. Table S1. RT-qPCR primers used in this study. Data S1. Lists of differentially expressed genes identified between wild type and *MiMIF-2* expressing Arabidopsis lines. Data S2. Lists of differentially expressed genes identified between wild type and *MiMIF-2* expressing Arabidopsis lines after treatment with flg22. Figure S1. ROS visualization in nematode infected Arabidopsis roots. CM-H_2_DCFDA staining of ROS in wild-type (WT) and *MiMIF-2* expressing (MiMIF-2-3 and MiMIF-2-4) roots following *M. incognita* infection. Images were captured by confocal microscopy (LeicaSP8) 1-day post inoculation (dpi). Micrographs A are images of differential interference contrast (grey). Micrographs B are images of GFP fluorescence (green). Micrographs C are overlays of images of the differential interference contrast and GFP-fluorescence. GFP, green fluorescence protein. Scale bar, 50 µm. N, nematode. Figure S2. H_2_O_2_ detection after co-expression MiMIF-2 and Bax in *N. benthamiana* leaves. H_2_O_2_ production in *N. benthamiana* leaves was assessed by DAB staining. *N. benthamiana* leaves were infiltrated with *Agrobacterium tumefaciens* carrying the corresponding constructs, and measurements were performed at 2 dpi. Three independent assays were conducted and at least 5 plants and/or 15 leaves were observed. Figure S3. The clusters of differential expression genes between wild type and *MiMIF-2* expressing Arabidopsis transcriptomes after treatment with flg22 24 h. (A) COG function classification of DEGs. DEGs were categorized into 21 function classes. Among them, signal transduction mechanisms (14.89%), secondary metabolites biosynthesis, transport and catabolism (10.51%), carbohydrate transport and metabolism (8.23%), posttranslational modification, protein turnover, chaperones (7.53%) and defense mechanisms (6.65%), amino acid transport and metabolism (5.78%), lipid transport and metabolism (5.6%) were relatively abundant. (B) GO terms classification of DEGs. DEGs were classified into three processes, including biological process, cellular component and molecular function.
