# Supplementary figures and images for: MiMIF-2 Effector of Meloidogyne incognita Exhibited Enzyme Activities and Potential Roles in Plant Salicylic Acid Synthesis
Source: Int J Mol Sci. 2020 May 15;21(10):3507. doi: 10.3390/ijms21103507 (PMC7278917; doi:10.3390/ijms21103507)

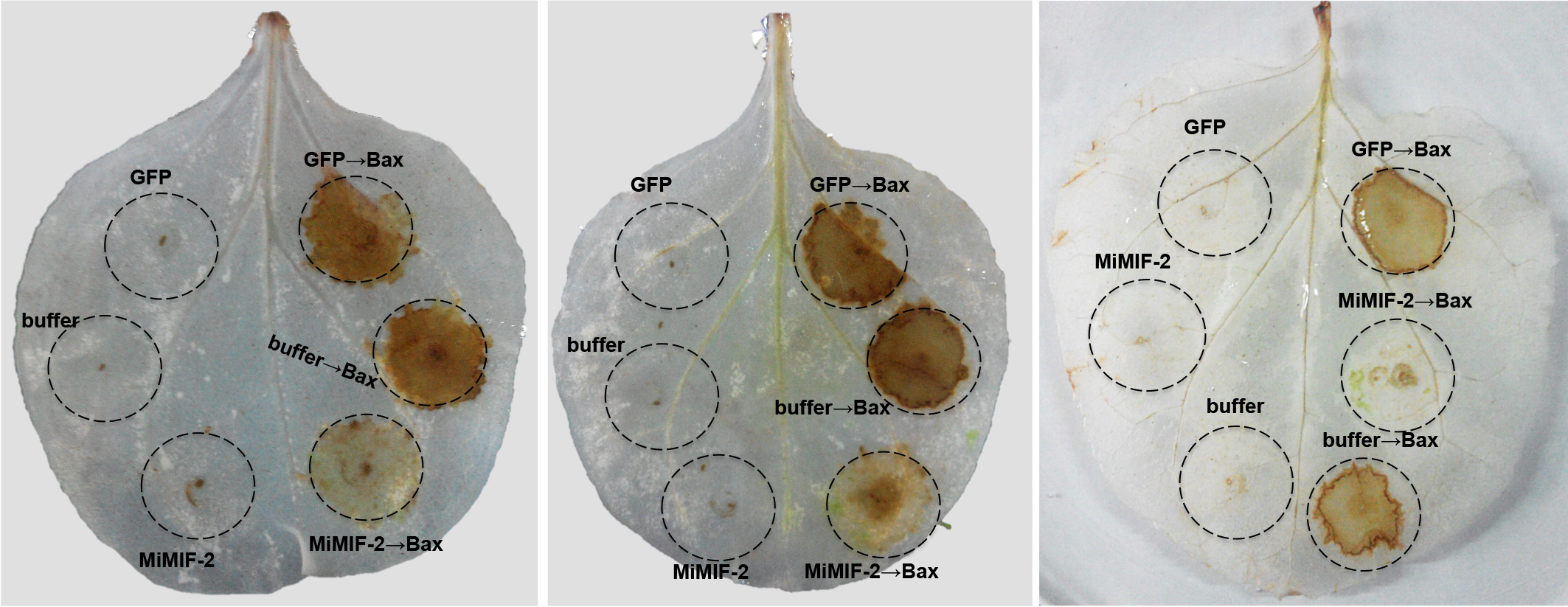

Supplement: Supplementary file 1 [file ijms-21-03507-s001.zip › Figure S2.tif]

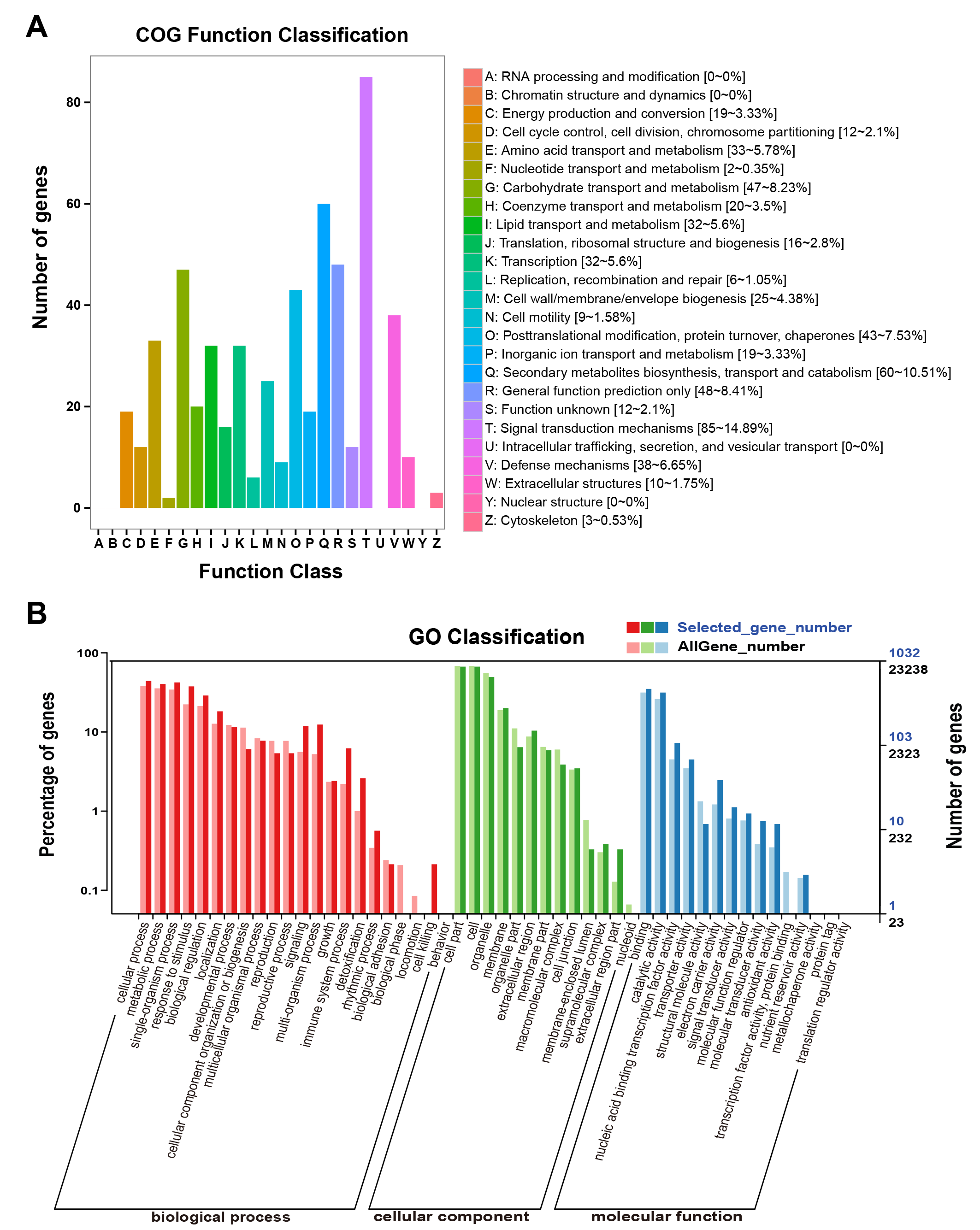

Supplement: Supplementary file 1 [file ijms-21-03507-s001.zip › Figure S3.tif]

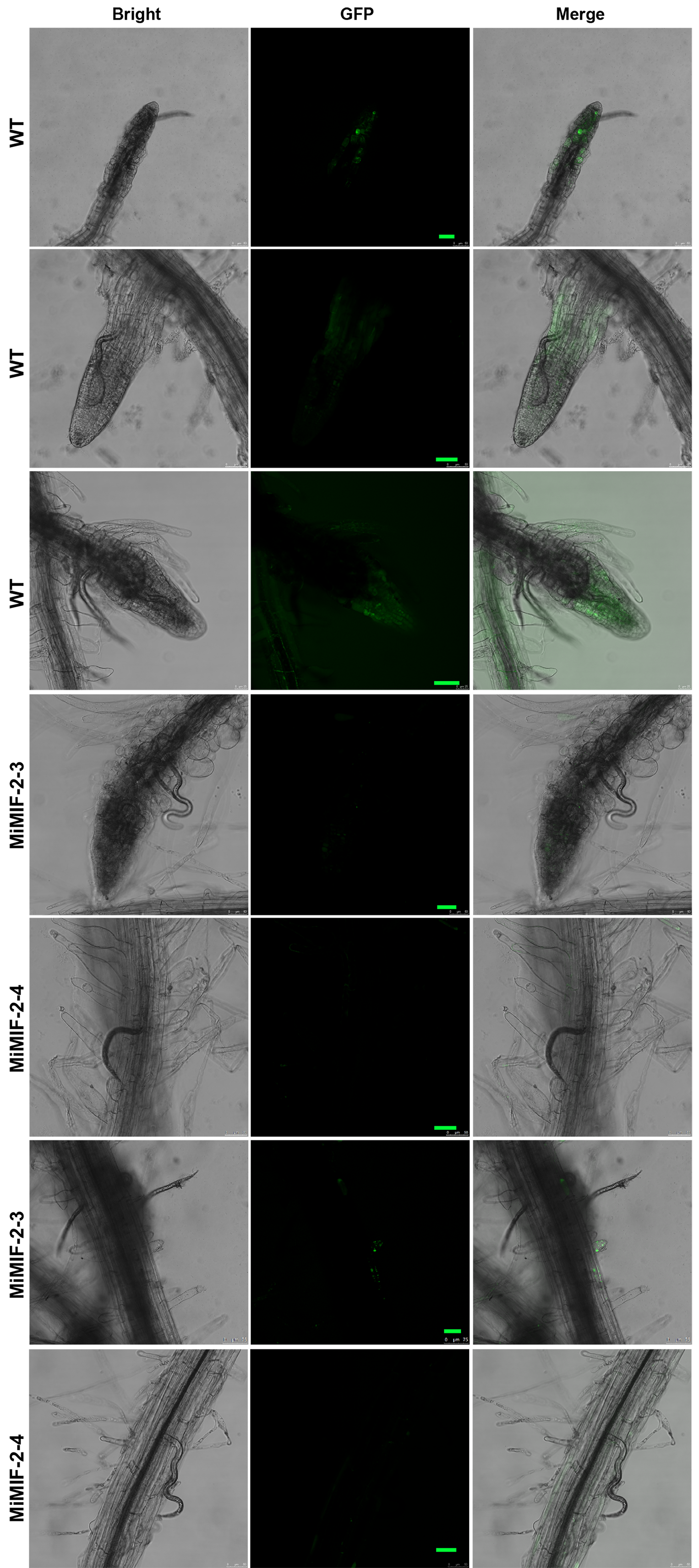

Supplement: Supplementary file 1 [file ijms-21-03507-s001.zip › Figure S1.tif]
